# Diagnosis, prevalence estimation and burden measurement in population surveys of headache: presenting the HARDSHIP questionnaire

**DOI:** 10.1186/1129-2377-15-3

**Published:** 2014-01-08

**Authors:** Timothy J Steiner, Gopalakrishna Gururaj, Colette Andrée, Zaza Katsarava, Ilya Ayzenberg, Sheng-Yuan Yu, Mohammed Al Jumah, Redda Tekle-Haimanot, Gretchen L Birbeck, Arif Herekar, Mattias Linde, Edouard Mbewe, Kedar Manandhar, Ajay Risal, Rigmor Jensen, Luiz Paulo Queiroz, Ann I Scher, Shuu-Jiun Wang, Lars Jacob Stovner

**Affiliations:** 1 Department of Neuroscience, Norwegian University of Science and Technology, Trondheim, Norway; 2 Department of Neuroscience, Imperial College London, London, UK; 3 Department of Epidemiology, Centre for Public Health, National Institute of Mental Health and Neurosciences, Bangalore, India; 4 Centre of Public Health Research, CRP-Santé, Strassen, Luxembourg; 5 Department of Pharmaceutical Sciences, University of Basel, Basel, Switzerland; 6 Department of Neurology, Evangelic Hospital, Unna, Germany; 7 University of Duisburg-Essen, Essen, Germany; 8 Department of Neurology, St Josef Hospital, Ruhr University Bochum, Bochum, Germany; 9 Department of Neurology, Chinese PLA General Hospital, Beijing, China; 10 King Abdullah International Medical Research Center, King Saud Ben Abdulaziz University for Health Sciences, National Guard Health Affairs, and Prince Mohammed Ben Abdulaziz Hospital, Ministry of Health, Riyadh, Saudi Arabia; 11 Department of Internal Medicine, Faculty of Medicine, Addis Ababa University, Addis Ababa, Ethiopia; 12 Department of Neurology, University of Rochester, Rochester, NY, USA; 13 Chikankata Hospital, Mazabuka, Zambia; 14 Department of Neurology, Baqai Medical University, Karachi, Pakistan; 15 Department of Mental Health and Clinical Psychiatry, Chainama College of Health Sciences, Lusaka, Zambia; 16 Dhulikhel Hospital, Kathmandu University Hospital, Dhulikhel, Kavre, Nepal; 17 Danish Headache Center, Department of Neurology, Glostrup Hospital, University of Copenhagen, 2600, Glostrup, Denmark; 18 Department of Neurology, Universidade Federal de Santa Catarina, Florianópolis, Brazil; 19 Preventive Medicine and Biometrics, Uniformed Services University, Bethesda, MD, USA; 20 Department of Neurology, Taipei Veterans General Hospital, Taipei, Taiwan; 21 Faculty of Medicine, National Yang-Ming University School of Medicine, Taipei, Taiwan; 22 Norwegian National Headache Centre, Norwegian University of Science and Technology and St Olavs University Hospital, Trondheim, Norway

**Keywords:** Epidemiology, Burden of headache, Methodology, Diagnostic instrument, Global Campaign against Headache

## Abstract

The global burden of headache is very large, but knowledge of it is far from complete and needs still to be gathered. Published population-based studies have used variable methodology, which has influenced findings and made comparisons difficult. The Global Campaign against Headache is undertaking initiatives to improve and standardize methods in use for cross-sectional studies. One requirement is for a survey instrument with proven cross-cultural validity. This report describes the development of such an instrument. Two of the authors developed the initial version, which was used with adaptations in population-based studies in China, Ethiopia, India, Nepal, Pakistan, Russia, Saudi Arabia, Zambia and 10 countries in the European Union. The resultant evolution of this instrument was reviewed by an expert consensus group drawn from all world regions. The final output was the Headache-Attributed Restriction, Disability, Social Handicap and Impaired Participation (HARDSHIP) questionnaire, designed for application by trained lay interviewers. HARDSHIP is a modular instrument incorporating demographic enquiry, diagnostic questions based on ICHD-3 beta criteria, and enquiries into each of the following as components of headache-attributed burden: symptom burden; health-care utilization; disability and productive time losses; impact on education, career and earnings; perception of control; interictal burden; overall individual burden; effects on relationships and family dynamics; effects on others, including household partner and children; quality of life; wellbeing; obesity as a comorbidity. HARDSHIP already has demonstrated validity and acceptability in multiple languages and cultures. Modules may be included or not, and others (*eg*, on additional comorbidities) added, according to the purpose of the study and resources (especially time) available.

## Introduction

The global burden of headache is very large [1-4]. The Global Burden of Disease Survey 2010 (GBD2010) confirmed that headache disorders are among the top 10 causes of disability worldwide [4], a finding foreseen by Stovner et al. several years earlier [3]. It is, paradoxically, a burden widely ignored [5]. The following conclusion appeared in the *Atlas of Headache Disorders and Resources in the World 2011*, published by the World Health Organization (WHO) [6]:

“The facts and figures [on headache] illuminate worldwide neglect of major causes of public ill-health, and the inadequacies of responses to them in countries throughout the world.”

Reduction of the burden of headache worldwide is the central purpose of the Global Campaign against Headache [1,2,7], conducted by *Lifting The Burden* (LTB), a UK-registered nongovernmental organization, in official relations with WHO. The Campaign’s objectives require action supported by awareness, the latter underpinned by knowledge. The knowledge base is evidence of the levels, nationally and worldwide, of headache-related ill-health and health-care need; it shows what manner of change – and how much – is required; it supports the humanitarian, economic and political arguments for change; and it signals the priority that should be accorded to action for change. The knowledge base is the foundation on which everything must be built; it needs to be complete, and sound. Unfortunately it is not.

The known epidemiology of headache disorders was collated in 2007 [3], the process revealing that prevalence was often studied without assessment of attributable burden, that rather little was known of disorders other than migraine and that there was a serious lack of studies of childhood and adolescent headache. Perhaps more tellingly than all of these, the very large geographical gaps – regions where very little or nothing at all was known of headache in the population – embraced more than half the people in the world. Many of these gaps remained when the evidence was adduced for GBD2010. Furthermore, wide variations in findings among the published studies were clearly influenced by their methodological differences [3,8].

The Global Campaign began to address these deficiencies several years ago, planning population-based studies in Georgia [9], India [10], China [11], Russia [12] and Pakistan [13], in Ethiopia, Guatemala, Nepal, Saudi Arabia and Zambia (in progress or analysis), and in Morocco, Egypt, Peru and Sri Lanka (planned), and providing intellectual support for the 10-country Eurolight project [14]. These major undertakings focused on the primary headache disorders, essentially migraine and tension-type headache (TTH), which have public-health importance by virtue of being common, ubiquitous, disabling and, to a large extent, treatable. Medication-overuse headache (MOH), secondary by definition [15], was always included because, on present understanding, it arises mostly if not entirely through mistreatment of these primary headache disorders. Unquestionably, MOH contributes to public ill-health; at individual level, it is more costly even than migraine [16].

In the course of planning these studies, *Lifting The Burden* and its collaborators developed both a standardized protocol and a survey instrument, and tested them empirically, the latter in multiple languages. Here we describe the genesis of the survey instrument, and its evolution into the Headache-Attributed Restriction, Disability, Social Handicap and Impaired Participation (HARDSHIP) questionnaire. Validation studies are reported elsewhere [11-13].

## Methods

The process was led by TJS and LJS who, with help from Dr Tarun Dua, Department of Mental Health and Substance Abuse, World Health Organization, conceived the first draft, suggested the areas of enquiry, proposed the question structure and phrasing and established a design template. As population-based studies were planned and undertaken, and in consultation with a wide range of experts and local investigators from over 20 countries, the instrument was amended, expanded through a process of item development and refined through item rejection based on empirical experience. Some questions were rephrased. The studies provided a continuing learning experience, fostering improvements through a series of plan-do-study-act cycles, and were a highly influential part of the development process.

During this process, the diagnostic questions were the subject of several validation studies, now completed in India [10] (translated into Kannada), China [11] (translated into Mandarin Chinese), Russia [12] (translated into Russian) and Pakistan [13] (translated into Urdu). Others are ongoing, including a study in Saudi Arabia using an Arabic translation. In each of these, questionnaire-derived diagnoses were compared with “gold-standard” diagnoses made by headache experts, in most cases in a subset of participants in a nationwide study.

In the context of a separate undertaking – to develop guidelines for the conduct and quality-assessment of population-based burden-of-headache studies – LJS and TJS convened an expert consensus group [8]. Members were selected with two considerations in mind: to include theoretical and/or practical experience and competence in headache epidemiology, and to ensure international and cross-cultural relevance. To the latter end, members were drawn from all six of the WHO world regions. This group (included among the authors) first acted as a sounding board and second, and very importantly, reviewed the structure, design and content of the instrument at a consensus meeting in Trondheim in September, 2011.

## Results

The HARDSHIP questionnaire (Additional file 1) is a structured questionnaire which may be administered by medical or (more usually) trained lay interviewers. It has a modular design: separate question sets cover demographic characteristics, screen for caseness (headache disorder present or not), diagnose headache type and address each of the several quantifiable components of burden. Headache occurring on ≥15 days/month, including MOH, is separated from episodic headache. The likelihood of multiple headache types occurring in a single respondent is recognized, and the potential confusion arising therefrom is minimized by asking him or her to identify, and focus upon, the one that is subjectively the most bothersome. Diagnostic questions based on the criteria of the International Classification of Headache Disorders, 3rd edition beta version (ICHD-3 beta) [17], and enquiries into burden, are directed at this headache type. Responses to the diagnostic questions are transformed into diagnoses algorithmically (Additional file 2); diagnoses are *not* made by the interviewer(s).

The diagnostic validation studies from India [10], China [11], Russia [12] and Pakistan [13] produced the results shown in Table 1. In all studies, sensitivity was better for migraine than for TTH.

**Table 1:** Sensitivities, specificities, positive (PPV) and negative (NPV) predictive values, and overall agreement with “gold-standard” diagnoses (kappa), of the diagnostic question set for migraine and tension-type headache

**Study**	**Migraine**					**Tension-type headache**				
	**Sensitivity**	**Specificity**	**PPV**	**NPV**	**Kappa**	**Sensitivity**	**Specificity**	**PPV**	**NPV**	**Kappa**
**India**[10]^1^	0.63	0.85	0.55	0.89	0.46	0.57	0.81	0.61	0.79	0.39
**China**[11]^2^	0.83	0.99	0.83	0.99	0.82	0.51	0.99	0.86	0.92	0.59
**Russia**[12]^3^	0.77	0.82	0.69	0.87	0.58	0.64	0.91	0.86	0.74	0.56
**Pakistan**[13]^4^	0.74	0.87	0.60	0.92	0.56	0.60	0.92	0.69	0.88	0.54

^1^Translated into Kannada; ^2^translated into Mandarin Chinese; ^3^translated into Russian; ^4^translated into Urdu.

Separate modules (each of which may be included or not according to study purpose, time constraints, resources available and cultural appropriateness) cover the following aspects of headache-attributed burden: symptom burden; health-care utilization; disability and productive time losses; impact on education, career and earnings; perception of control; interictal burden; overall individual burden (as willingness to pay for treatment); effects on relationships, love life and family dynamics; effects on others, including household partner and children; quality of life; wellbeing; obesity as a comorbidity.

## Discussion

Demographic enquiry is essential to characterize the sample. Data are needed in order to compare those who have been selected with the population of interest from whom they are drawn and of whom they are intended to be representative. While, ideally, these data will reflect all factors that may influence prevalence and/or burden of headache, this objective is necessarily limited by the availability of data characterizing the entire population. National (but perhaps not regional) statistics are commonly available for gender and age distributions. Even when they are not, these are of such prime importance in headache epidemiology that they must be known in the sample. Social situation (especially wealth), habitation (urban or rural) and ethnicity and/or culture may be important influencers of prevalence or burden, and are therefore of some interest.

Diagnosis must follow ICHD criteria [17] because these are the common language of definition and description of headache disorders [18]. There is no alternative, even though ICHD criteria were not designed for epidemiological enquiry and are not particularly well-suited to it [18]. For epidemiological purposes, these criteria must be built into a structured questionnaire, although this is not how diagnoses are usually made in clinical settings. Open questions are difficult to interpret and categorize, and do not permit algorithmic determination.

Certain criteria distinguishing between migraine and TTH pose particular problems in population surveys [18]. First, empirically it has been found difficult to gather correct responses on headache duration [3], requiring patients to consider *untreated* attacks, which they may never have or last had long ago. This results in a high proportion of probable diagnoses because duration criteria appear unfulfilled [18]. Second, there are no easy lay explanations of photo- and phonophobia, which are technical concepts, and even more difficult is to specify what degrees of photo- and phonophobia fulfil migraine criteria in ICHD [18]. False-positive responses, more likely when answers are forced (without the response option of “don’t know”, which is diagnostically unhelpful), push diagnoses towards migraine. HARDSHIP includes a “not sure” option and applies a rule that “not sure” implies absence of the symptom. The reasoning is that presence of a symptom creates a definite awareness of it, and only its absence allows uncertainty. This suggested approach requires further empirical testing.

MOH is diagnosable in cross-sectional studies only as an association of medication overuse with frequent headache (there is no evidence available of causation) [18]. Therefore, all such cases are probable MOH, and it is important to recognize this limitation during analyses and interpretation.

Symptoms of common headache disorders include pain, and, of migraine, nausea, vomiting and photo- and/or phonophobia. Symptom burden is addressed in HARDSHIP by questions 14, 15, 20, 21/23, 24, 29–32, 36 and 37. Pain can be quantified at individual level as a product of intensity, frequency and duration, and at population level as the product of the average among individuals and prevalence. Nausea, photophobia and phonophobia are almost impossible to quantify, but their occurrence can be recorded and frequencies expressed.

Disability attributed to headache is also difficult to quantify completely. Common proxies are lost time and reduced productivity, for which well-validated instruments exist [19,20]. HARDSHIP (questions 38–44 and 58–62) imports the Headache-Attributed Lost Time (HALT) index [20]. Reliability of recall is an issue here. Burden questions have commonly been limited to a 3-month timeframe [19,20] as a compromise between the limits of recall and the purpose of enquiry. When the latter is the assessment of an individual patient for therapeutic reasons, the period must be long enough to be representative of that individual. In large-group studies, this is quite unnecessary: different considerations apply, because population- rather than individual-representativeness is sought. Variations of HALT that record over shorter timeframes of one month (HALT-30) and one week (HALT-7) are being tested empirically [21].

Enquiry into headache yesterday (effectively HALT-1) (HARDSHIP questions 34–45) avoids recall problems almost altogether [18,22]. It cannot describe the proportion of the population with an active headache disorder, but it yields very accurate information on burden in each individual and, potentially, a rather precise estimate of population burden on a particular day and, therefore, on any day (assuming no major seasonal variation). A large sample is necessary, because 1-day prevalence of episodic headache disorders is obviously much lower than 1-year prevalence. This module probably should not be used except in an unscheduled interview (face-to-face or telephone) [18]; if it is received by a person with headache on that day, he or she may well postpone answering it until their next headache-free day.

Interictal burden (HARDSHIP questions 64–66) arises because headache attacks are unpleasant, and those who experience them frequently are likely to worry about when the next may occur, and/or attempt to eliminate possible triggers through lifestyle compromise. Interictal burden, which is continuous, is likely to affect subjective wellbeing and may be sufficient to impair quality of life. It is perhaps adequately, if not specifically, captured by measures of subjective wellbeing and quality-of-life measures. HARDSHIP imports, as modules, WHOQoL-8 [23] (questions 90–97) and the four questions on subjective wellbeing taken from the UK-ONS 2012 survey [24] (98–101).

Cumulative burden (HARDSHIP questions 51–57), accruing over a lifetime, cannot be fully assessed until late in a lifetime. Furthermore, attribution may be uncertain. Nevertheless, a consequence of recurring inability to work may be decreased probability of promotion, and a consequence of lost school-time may be reduced career opportunities. These may be heavy burdens.

An overall summary measure of individual burden is unlikely to be comprehensive, but the concept is attractive for its simplicity [18]. One such measure is willingness-to-pay (WTP) (HARDSHIP questions 67–74). Its reliability as a burden measure remains unclear: its hypothetical nature allows a potential disconnection between what respondents *say* they will pay and what they actually *will* pay when confronted by the reality, and of course WTP is constrained by ability to pay. Nevertheless, this form of enquiry has been used to assess sustainability of health-care initiatives in resource-poor countries [25].

Burden on others, unaffected by headache themselves, is addressed by HARDSHIP questions 75–86. Subjective interpretations are unavoidable. A full account necessitates enquiries among the others, which in practical terms may be possible only among close family members.

Health-care resource consumption (HARDSHIP questions 45–50) is relatively easy to enquire into, but subject to recall bias. It should also be easy to establish who pays for it (the patient, employer, insurer or society via the State). It is less easy to attach accurate costs to individual items of health care, and this may necessitate separate research into health-care costs in the country or region in question [16].

By far the greater part of the financial cost of headache is the indirect cost of absenteeism and reduced effectiveness at work [16,26] (HARDSHIP questions 58 and 59). This cost may be borne by individuals, but commonly falls upon employers and/or insurers, and is a cost to national economies (societal economic burden).

Enquiry into comorbidity includes body mass index (HARDSHIP questions 87–89), since obesity may be an important and potentially remediable risk factor for frequent headache [27]. Other (for example, psychiatric) comorbidities can be included by bringing in other instruments (*eg*, the Hospital Anxiety and Depression Scale [28], or the Shona Symptom Questionnaire [29], which may be better suited to some cultures).

The strengths of HARDSHIP are several. First, a very broad base of expert opinion contributed to its evolution. Second, it has undergone testing in many cultures and settings: so far in 19 countries and 18 languages. In some countries within the Eurolight study [14], rather than being administered by interviewer as envisaged in its development, it was mailed or handed out for self-completion. Third, its successful employment in multiple studies has built a collection of studies conducted with similar methodology in different world regions, facilitating inter-regional comparisons. Likewise, its use in future studies will enable further comparisons. Fourth, its modular design renders it highly amenable to adaptation to suit purpose, resource availability (especially time) and cultural sensitivities.

The one known limitation, discovered empirically, is that the diagnostic question set is relatively insensitive to TTH in all languages and cultures in which it has been tested. The problem is attributable partly to these questions being necessarily tied to ICHD, which makes it difficult to resolve because there is very limited scope for change. More particularly, though, it is due to the nature of TTH itself. Being usually a mild-to-moderate headache, TTH is more likely than migraine to go unreported. Beyond this, because it lacks specific features, it is diagnosed through absence of the features characteristic of migraine, and therefore effectively by default. Reduced sensitivity is inevitable in such circumstances. In this context it is worth noting that, uniquely among the so far reported studies using HARDSHIP, the population survey in China was conducted by physicians. For migraine, the diagnostic question set performed best in China (Table 1) but, for TTH, sensitivity remained low.

In the studies conducted using HARDSHIP, migraine prevalence has been high, although not in China (1-year prevalence 9.3% [30]), where headache generally appears less prevalent. In Russia the reported 1-year prevalence is 20.8% [31]; in India (Karnataka State) it is over 25% [unpublished]; in other countries not yet reported, levels of this order have been found. There are two possible explanations of this. First is that some cases of TTH are incorrectly diagnosed as migraine, a possibility suggested by the low sensitivity to TTH just referred to. Second is that these findings reflect the truth, and that migraine is more common than has been thought (global 1-year prevalence about 11% [3]). We suggest that careful enquiry observes a higher prevalence of migraine by capturing milder cases (*ie*, by better case ascertainment), and that, although both of these explanations may contribute in part, migraine is indeed more prevalent than past estimates have suggested. Alstadhaug et al. [32] reported the prevalence of migraine in Norwegian neurologists, among whom diagnoses should be correct and case ascertainment very high. The age-adjusted 1-year prevalence of migraine headache (*ie*, excluding cases of aura only) was 26.3% (95% CI: 18.5-34.2%), 15.9% in males and 36.7% in females. It is unlikely that Norwegian neurologists are biologically unique.

## Conclusions

For better and comparable population-based studies of the burden of headache, there is a clear need for a survey instrument with proven cross-cultural validity, adaptable to the circumstances of particular studies and resource-availability [8]. HARDSHIP has demonstrated validity and acceptability in multiple languages and cultures. Modules may be included or not, and others (*eg*, on comorbidities) added, according to the purpose(s) of the study.

## Abbreviations

CI: Confidence interval; GBD2010: Global burden of disease survey 2010; HALT: Headache-attributed lost time; HARDSHIP: Headache-attributed restriction, disability, social handicap and impaired participation; ICHD: International classification of headache disorders; LTB: *Lifting The Burden*; MIDAS: Migraine disability assessment; MOH: Medication-overuse headache; TTH: Tension-type headache; WHO: World Health Organization; WTP: Willingness to pay.

## Competing interest

TJS, GLB, RJ, ZK and LJS are the directors and trustees of *Lifting The Burden*. GLB, TJS and LJS served as experts in the Global Burden of Disease Survey 2010. GLB has received research funds from the US National Institutes of Health and the Dana Foundation.

## Authors’ contributions

TJS and LJS created the original concept and assembled the group of experts. All authors were members of the expert consensus group or engaged in developing the questionnaire in the context of population-based studies, or both. TJS drafted the manuscript. All authors reviewed the manuscript in various drafts and approved the final version.

## Supplementary Material

Additional file 1 The HARDSHIP questionnaire. Click here for file

Additional file 2 Diagnostic algorithm. Click here for file
