# Supplementary material for: Diagnosis, prevalence estimation and burden measurement in population surveys of headache: presenting the HARDSHIP questionnaire
Source: J Headache Pain. 2014 Jan 8;15(1):3. doi: 10.1186/1129-2377-15-3 (PMC3906903; doi:10.1186/1129-2377-15-3)
Supplement: Additional file 1 — The HARDSHIP questionnaire. [file 1129-2377-15-3-S1.doc]

**Additional file 1 The HARDSHIP questionnaire**

| ***Lifting The Burden***  **in Official Relations with the World Health Organization**  **The Global Campaign against Headache**  **Headache-attributed restriction, disability, social handicap and impaired participation (HARDSHIP) questionnaire**  **for administration by medical or trained lay interviewers  to population samples** |
| --- |

| **Centre identifier**  (to be completed by the centre) | | | | | **_____________________________** | | | | | | | | | | | | | | | | | | | | | | |
| --- | --- | --- | --- | --- | --- | --- | --- | --- | --- | --- | --- | --- | --- | --- | --- | --- | --- | --- | --- | --- | --- | --- | --- | --- | --- | --- | --- |
| **Participant identifier**  (to be completed by the interviewer) | | | | | | | | | | | | | | | | | | | | | | | | | | | |
| □  enter letter to identify stratum:  U: urban  S: semi-rural  R: rural | | □□□ □□□  from master lists of sampling units and households:  enter 3-digit number to identify sampling unit followed by 3-digit number to identify household within sampling unit | | | | | | | | | | | | | | | | | | | | □□  from occupant list on next page:  enter 2-digit number to identify household occupant | | | | | |
| **Interviewer identifier**  (to be completed by the interviewer)  ______________________________ | | | | | **Interviewer signature (on completion):** | | | | | | | | | | | | | | | | | | | | | | |
| **Participant identification** | | | | | | | | | | | | | | | | | | | | | | | | | | | |
| Address of household and name of head of household  [not required if the survey data are to remain anonymous] | | |  | | | | | | | | | | | | | | | | | | | | | | | | |
| Numbered list of household occupants  (enter given name, age and gender of each occupant in the order supplied)  (age may be estimated if the date of birth is unknown) | | |  | | | **Given name** | | | | | | | | | | | | | | | | **Age (y)** | | | | | **M/F** |
|  |  |  | **01** | | |  | | | | | | | | | | | | | | | |  | | | | |  |
|  |  |  | **02** | | |  | | | | | | | | | | | | | | | |  | | | | |  |
|  |  |  | **03** | | |  | | | | | | | | | | | | | | | |  | | | | |  |
|  |  |  | **04** | | |  | | | | | | | | | | | | | | | |  | | | | |  |
|  |  |  | **05** | | |  | | | | | | | | | | | | | | | |  | | | | |  |
|  |  |  | **06** | | |  | | | | | | | | | | | | | | | |  | | | | |  |
|  |  |  | **07** | | |  | | | | | | | | | | | | | | | |  | | | | |  |
|  |  |  | **08** | | |  | | | | | | | | | | | | | | | |  | | | | |  |
|  |  |  | **09** | | |  | | | | | | | | | | | | | | | |  | | | | |  |
|  |  |  | **10** | | |  | | | | | | | | | | | | | | | |  | | | | |  |
|  |  |  | **11** | | |  | | | | | | | | | | | | | | | |  | | | | |  |
|  |  |  | **12** | | |  | | | | | | | | | | | | | | | |  | | | | |  |
|  |  |  | **13** | | |  | | | | | | | | | | | | | | | |  | | | | |  |
|  |  |  | **14** | | |  | | | | | | | | | | | | | | | |  | | | | |  |
| Select one occupant at random from the total number of occupants: the selected person will be the participant.  Enter the number in the next column and on the previous page. | | | | | | | | | | | | | | | | | | | | | | □□  enter number to identify selected household occupant | | | | | |
| Thank you for answering the following questions. Please begin by entering **today’s date**, and then answer **all questions** **on this day**. | | | | | | | | | | | | | | | | | | | | | | | | | | | |
| **1** | Please enter today’s date | | | | | | | | | | | **_____/_____/________** | | | | | | | | | | | | | | | |
| **Demographic questions** | | | | | | | | | | | | | | | | | | | | | | | | | | | |
| **2** | What is your age? | | | | | | | | | | | **_____ years** | | | | | | | | | | | | | | | |
| **3** | What is your gender? (please tick one box) | | | | | | | | | | | **male** □ **female** □ | | | | | | | | | | | | | | | |
| **Social situation questions** | | | | | | | | | | | | | | | | | | | | | | | | | | | |
| **4** | What is your marital status?  (please tick one box **only**) | | | □  **single** | | | | | | □  **married** | | | | | | □  **widow or widower** | | | | | | | □  **separated or divorced** | | | | |
| **5** | Are you living with a household partner?  (please tick one box)  (a household partner may be husband or wife, or an unmarried partner of either gender in a stable relationship) | | | | | | | | | | | **no** □ **yes** □ | | | | | | | | | | | | | | | |
| **6** | Which of these is closest to your personal situation?  (please tick one box **only**) | | | □  **employed or self-employed** (go to question 7) | | | | | | | | | | | | | | | | | | | | | | | |
|  |  |  |  | □  **homemaker or housewife** (go to question 8) | | | | | | | □  **student** (go to question 8) | | | | | □  **unemployed** (go to  question 8) | | | | | | | □  **retired** (go to question 8) | | | | |
| **7** | Which of these best describes your work?  (please tick one box **only**)  [the categories listed are suggestions; they should be adapted and/or supplemented as appropriate for the country] | | | | | | | | | | | **professional**  **semi-professional**  **skilled worker**  **semi-skilled worker**  **unskilled worker** | | | | | | | | | | | □  □  □  □  □ | | | | |
| **8** | What is your **total net household income** per year? (please tick one box)  [the values of W, X, Y and Z in national currency units (NCU) should correspond to the national household income quintiles, so that one fifth of the population falls into each income category; as an alternative, the question may relate to personal income and W, X, Y and Z should then correspond to national per capita income quintiles] | | | | | | | | | | | **less than NCU W**  **between NCU W+1 and NCU X**  **between NCU X+1 and NCU Y**  **between NCU Y+1 and NCU Z**  **more than NCU Z** | | | | | | | | | | | □  □□  □  □ | | | | |
| **9** | How many years did you **complete** in **full-time education**? (please add together all the years at school or places of higher education) | | | | | | | | | | | **______ years** | | | | | | | | | | | | | | | |
| **10** | What is your native language (the language you first learned to speak)? | | | | | | | | | | | **enter name of language:** | | | | | | | | | | | | | | | |
| **11** | What language do you usually speak in your own home?  [this question may, if appropriate, be replaced or supplemented by questions on ethnicity] | | | | | | | | | | | **enter name of language:** | | | | | | | | | | | | | | | |
| **Screen questions** | | | | | | | | | | | | | | | | | | | | | | | | | | | |
| **12** | Have you **ever** had a headache **in your lifetime**?  (please tick one box) | | | | | | | | | | | **no** □ **yes** □ | | | | | | | | | | | | | | | |
| **13** | Have you had a headache **during the last 12 months**?  (please tick one box) | | | | | | | | | | | **no** □ **yes** □  (if no, go directly to question 87) | | | | | | | | | | | | | | | |
| **14** | During **the last 30 days**, on how many of these days did you have a headache?  (please enter number of days between 0 and 30) | | | | | | | | | | | **______ days**  (if you answered between 15 and 30 days, please continue with question 15; otherwise, go directly to question 19) | | | | | | | | | | | | | | | |
| **“Daily” headache questions**  You have said that you had headache **on 15 or more days in the last month**. Please think about these headaches. | | | | | | | | | | | | | | | | | | | | | | | | | | | |
| **15** | How long do these headaches usually last?  (please enter the number of minutes or hours, or tick the box) | | | | | | | | | | | **____ min or ____ hr** | | | | | | | | | | | □  **never goes away** | | | | |
| **16** | Do you take **any medication to treat** these headaches?  (please tick one box)  (please note that this question is about treatment to **relieve** the headache, not daily treatment to **prevent** headache) | | | | | | | | | | | **no** □ **yes** □  (if no, go directly to question 19) | | | | | | | | | | | | | | | |
| **17** | What medication **do you use most** to treat these headaches?  and what other medications do you also take for this purpose?  (if there are no others, please write “none”)  (please note that this question is only about treatment to **relieve** headache) | | | | | | | | | | | **name the most-used medication:**  **list all other medications:** | | | | | | | | | | | | | | | |
| **18** | Altogether, on how many days in the last 30 days did you take these medications?  (please enter number of days between 0 and 30) | | | | | | | | | | | **______ days** | | | | | | | | | | | | | | | |
| **“Most bothersome headache” questions**  These are questions on the headaches that interfere most with your life. These headaches may be the same as the headaches you have just described, or they may be different headaches if you have more than one type of headache. | | | | | | | | | | | | | | | | | | | | | | | | | | | |
| **19** | Please think about your headaches. Do you think they **are all of one type**, or are they of **more than one type**?  (please tick one box) | | | | | | | | | | | **one** □ **more than one** □ | | | | | | | | | | | | | | | |
| If you answered one, the next questions are to diagnose this headache. Please start at question 20.  If you answered more than one, from now on please focus upon the headache type that on the whole bothers you most (*ie*, interferes most with your life).  The next series of questions are intended to diagnose this type of headache. Please start at question 20. | | | | | | | | | | | | | | | | | | | | | | | | | | | |
| **Diagnostic questions** | | | | | | | | | | | | | | | | | | | | | | | | | | | |
| **20** | How often do you have **this type of headache**?  (please tick box or enter the number of days per month or per year) | | | | | | | | | | | □  **every day** | | | | | | _____  **days/month** | | | | | _____  **days/year** | | | | |
| **21** | How long does **this type of headache** usually last?  (please enter the number of minutes, hours or days, or tick the box)  (if the headache goes away during sleep, count the time until you wake up without it) | | | | | | | | | | | **___ mins, ___ hours or ___ days**  **never goes away** □ | | | | | | | | | | | | | | | |
| **22** | Is your last answer **with or without** medication?  (please tick one box) | | | | | | | | | | | **with** □ **without** □  (if you answered “without medication”, please go to question 24) | | | | | | | | | | | | | | | |
| **23** | How long would it last **if you did not take medication**?  (please enter the number of minutes, hours or days) | | | | | | | | | | | **___ mins, ___ hours or ___ days** | | | | | | | | | | | | | | | |
| **24** | How bad is **this type of headache** usually?  (please tick one box) | | | | | | | | | | | □  **not bad** | | | | | | □  **quite bad** | | | | | □  **very bad** | | | | |
| **25** | There are many ways of describing a headache, but most are either throbbing or pressing.  Thinking still of **this type of headache**, which best describes the pain?  (please tick one box) | | | | | | | | | | | □  **throbbing  or pulsating**  (this means varying in  time with the heart beat) | | | | | | | | | | | □  **pressing, squeezing  or  tightening** | | | | |
| **26** | Is the pain of **this type of headache** usually on only one side of the head?  (please tick one box) | | | | | | | | | | | **no** □ **yes** □ | | | | | | | | | | | | | | | |
| **27** | Does exercise (like walking or climbing stairs) tend to make it worse?  (please tick one box) | | | | | | | | | | | **no** □ **yes** □ | | | | | | | | | | | | | | | |
| **28** | Thinking still of **this type of headache**, how does it affect your ability to do day-to-day activities?  (please tick one box) | | | | | | | | | | | □  **can do everything  as normal** | | | | | | □  **cannot do some things** | | | | | □  **can do nothing** | | | | |
| **29** | With **this type of headache**, do you usually feel nauseated (as though you may vomit or throw up)?  (please tick one box) | | | | | | | | | | | **no** □ **yes** □ | | | | | | | | | | | | | | | |
| **30** | With **this type of headache**, do you usually actually vomit (throw up)?  (please tick one box) | | | | | | | | | | | **no** □ **yes** □ | | | | | | | | | | | | | | | |
| **31** | When you have **this type of headache**, does daylight or other lighting bother you? In other words, do you prefer to be in the dark?  (please tick one box) | | | | | | | | | | | □  **no** | | | | | | □  **not sure** | | | | | □  **yes** | | | | |
|  |  |  |  |  |  |  |  |  |  |  |  | (this question refers to ordinary levels of light, not bright lighting) | | | | | | | | | | | | | | | |
| **32** | When you have **this type of headache**, does noise bother you? In other words, do you prefer to be in the quiet?  (please tick one box) | | | | | | | | | | | □  **no** | | | | | | □  **not sure** | | | | | □  **yes** | | | | |
|  |  |  |  |  |  |  |  |  |  |  |  | (this question refers to ordinary levels of noise, not very loud noise) | | | | | | | | | | | | | | | |
| **33** | Has a **health-care professional** ever given you a diagnosis for **this type of headache**?  (please tick one box and, if yes, enter the diagnosis) | | | | | | | | | | | **no** □ **yes** □  **If yes, please write the diagnosis:** | | | | | | | | | | | | | | | |
| The next series of questions are specifically about **yesterday** (the day before you fill in your answers).  It is very important that the answers you give are about **yesterday** and not any other day. | | | | | | | | | | | | | | | | | | | | | | | | | | | |
| **Questions about yesterday** | | | | | | | | | | | | | | | | | | | | | | | | | | | |
| **34** | Did you have a **headache yesterday**?  (please tick one box) | | | | | | | | | | | **no** □ **yes** □  (if no, go directly to question 46) | | | | | | | | | | | | | | | |
| **35** | Was this the type of headache you have just been describing?  (please tick one box) | | | | | | | | | | | **no** □ **yes** □ | | | | | | | | | | | | | | | |
| **36** | Please think about the **headache you had yesterday**. How long did it last?  (please tick the box if it was present all day, from waking in the morning until bedtime, or enter the number of hours between 1 and 24) | | | | | | | | | | | **all day** □ **or _____ hours** | | | | | | | | | | | | | | | |
| **37** | How bad was this **headache yesterday**?  (please tick one box) | | | | | | | | | | | □  **not bad** | | | | | | □  **quite bad** | | | | | □  **very bad** | | | | |
| **38** | Please think about everything you wanted to do **yesterday** if you had not had a headache.  How much of this did you **actually do**?  (please tick one box) | | | | | | □  **nothing** | | | | | □  **less than half** | | | | | | □  **more than half** | | | | | □  **everything** | | | | |
| **39** | Was **yesterday** a workday (either at your job or at school)?  (please tick one box) | | | | | | | | | | | **no** □ **yes** □  (if no, go directly to question 43) | | | | | | | | | | | | | | | |
| **40** | Because of your headache, did you miss work or school **yesterday**?  (please tick one box or enter the number of hours lost from work or school) | | | | | | □  **no** | | | | | **arrived late, took time out during the day or left early** (please enter the total number of hours lost)**:**  **________ hours** | | | | | | | | | | | □  **missed the whole day**  (please go to question 42) | | | | |
| **41** | If you were at work or school with your **headache yesterday**, how much of your work did you get done?  (please tick one box)) | | | | | | □  **nothing** | | | | | □  **less than half** | | | | | | □  **more than half** | | | | | □  **everything**  (please go to question 43) | | | | |
| **42** | Will you able to make up for this today or later?  (please tick one box) | | | | | | | | | | | □  **no** | | | | | | □  **partly** | | | | | □  **completely** | | | | |
| **43** | Please think about household work or general chores that you wanted to do **yesterday** if you had not had headache.  How much of this did you **actually do**?  (please tick one box) | | | | | | □  **nothing** | | | | | □  **less than half** | | | | | | □  **more than half** | | | | | □  **everything** | | | | |
| **44** | Please think about leisure and social activities that you wanted to do **yesterday** if you had not had headache.  How much of this did you **actually do**?  (please tick one box) | | | | | | □  **nothing** | | | | | □  **less than half** | | | | | | □  **more than half** | | | | | □  **everything** | | | | |
| **45** | What treatment did you take for the **headache you had yesterday**?  Please tick the box if you took nothing; otherwise, please list the names of all medications taken for headache yesterday, and the number of times each was taken yesterday. | | | | | | **nothing at all**  **List medications:**  (please list medications for **headache**, not for any other illnesses)  **________________________**  **________________________**  **________________________**  **________________________**  **________________________** | | | | | | | | | | | | | | | | □  **how many times you took each**  **_____**  **_____**  **_____**  **_____**  **_____** | | | | |
| **Health care questions**  The aim of the following questions is to help us know how much health care should be available to meet the needs of people with headache. | | | | | | | | | | | | | | | | | | | | | | | | | | | |
| **46** | Many different medications may be used successfully to treat headache.  Some are prescription-only, whilst others can be bought over the counter.  Please look at these lists. Which of these have you used **in the last month?**  Please tick the box if you took nothing at all in the whole of the last month; otherwise, enter by each medication the number of days on which you used it in the last month.  [This question is country-specific, and the list should be adapted as appropriate] | | | | | | | **nothing at all**  **almotriptan (Almogran)**  **eletriptan (Relpax)**  **frovatriptan (Migard)**  **naratriptan (Naramig)**  **rizatriptan (Maxalt)**  **sumatriptan (Imigran)**  **zolmitriptan (Zomig)**  **ergotamine (Cafergot, Migril)**  **domperidone (Motilium)**  **metoclopramide (Maxolon, Primperan)**  **aspirin (acetylsalicylic acid)**  **diclofenac (Voltarol)**  **ibuprofen (Nurofen)**  **ketoprofen (Ketocid, Orudis)**  **mefenamic acid (Ponstan)**  **naproxen (Naprosyn)**  **paracetamol (Panadol)**  **tolfenamic acid (Clotam)**  **Proprietary combination drugs:**  **Excedrin**  **Migraleve**  **Migramax**  **Nuromol**  **Paramax**  **Solpadeine**  **Syndol** | | | | | | | | | | | | | | | □  **number of days**  **_____**  **_____**  **_____**  **_____**  **_____**  **_____**  **_____**  **_____**  **_____**  **_____**  **_____**  **_____**  **_____**  **_____**  **_____**  **_____**  **_____**  **_____**  **_____**  **_____**  **_____**  **_____**  **_____**  **_____**  **_____** | | | | |
| **46 (cont)** | Are there any other medications you have used to treat your headache **in the last month**?  Please enter the name of each other medication and, by each, the number of days on which you used it in the last month. | | | | | | | **Name(s) of medication(s):**  (please list medications for **headache**, not for any other illnesses)  **________________________**  **________________________**  **________________________**  **________________________**  **________________________**  **________________________** | | | | | | | | | | | | | | | **number of days**  **_____**  **_____**  **_____**  **_____**  **_____**  **_____** | | | | |
| **47** | Medications to **prevent** headaches are usually taken daily. Are you taking any of these now?  Please enter the name(s) and, by each one, for how long in weeks or months you have been taking it. | | | | | | | **Name(s) of medication(s):**  **________________________**  **________________________**  **________________________** | | | | | | | | | | | | | | | **how long taken?**  **_____**  **_____**  **_____** | | | | |
| **48** | Many people with headache treat themselves, but others need professional advice.  Have you had professional advice about your headaches **in the last year**? Who from, and how many times?  Please tick all boxes that apply and, for each ticked box, enter the number of times in the last year.  [Other categories may be added or substituted when relevant to the country] | | | | | | | **no-one**  **nurse**  **physical therapist (physiotherapist, osteopath, chiropractor)**  **clinical officer**  **primary-care doctor (GP)**  **headache specialist**  **ear, nose and throat doctor**  **eye doctor**  **hospital emergency room**  **other (please specify):**  **__________________** | | | | | | | | | | | | | | □  □  □  □  □  □  □  □  □  □ | | **number of times**  **_____**  **_____**  **_____**  **_____**  **_____**  **_____**  **_____**  **_____**  **______** | | | |
| **49** | Most people with headache do not require any investigations, but occasionally these tests are done.  Because of your headaches, have you had any of these tests **in the last year**?  (please tick all that apply)  [Other country-relevant investigations, such as blood smear for malaria, may be added] | | | | | | | | | | | | | | **MRI brain scan**  **CT brain scan**  **x-rays of the neck**  **eye tests  (for glasses)**  **blood tests** | | | | | | | | □  □  □  □  □ | | | | |
| **50** | Have you, **in the last year**, been admitted to hospital **because of your headaches**?  (please tick one box and, if yes, enter the total number of days in hospital) | | | | | | | | | | | | | | **no** □ **yes** □  **total number of days _____** | | | | | | | | | | | | |
| **Impact questions**  The next questions are about the effects your headaches have on **your own life**. | | | | | | | | | | | | | | | | | | | | | | | | | | | |
| **51** | Have your headaches interfered with your **education**?  (please tick all boxes that apply **because of your headaches**) | | | | | □  **no** | | | | | | □  **yes, I did less well** | | | | | | □  **yes, I did not attempt something** | | | | | □  **yes, I gave up early** | | | | |
| **52** | Do you believe your headaches have made you less successful in your career?  (please tick all boxes that apply **because of your headaches**)  (if this question is not applicable to you, please tick no and go directly to question 54) | | | | | | | | | | | | | | **no**  **yes, I have done less well**  **yes, I have attempted less**  **yes, I have taken an easier job**  **yes, I have taken long-term sick leave**  **yes, I have retired early**  **yes, I am on a disability pension** | | | | | | | | | □  □  □  □  □  □  □ | | | |
| **53** | Have your headaches reduced your **earnings**?  (please tick one box) | | | | | | | | | | | **no** □ **yes** □ | | | | | | | | | | | | | | | |
| **54** | Do you feel that your employer and work colleagues understand and accept your headaches?  (please tick one box) | | | | | | | | | | | □  **no** | | | | | | | □  **partly** | | | | | □  **yes, fully** | | | |
| **55** | Do you feel that your family and friends understand and accept your headaches?  (please tick one box) | | | | | | | | | | | □  **no** | | | | | | | □  **partly** | | | | | □  **yes, fully** | | | |
| **56** | Do you avoid telling people that you have headaches?  (please tick one box) | | | | | | | | | | | **no** □ **yes** □ | | | | | | | | | | | | | | | |
| **57** | Taking into account everything you do to treat your headaches, how well do you think you control them?  (please tick one box) | | | | | | □  **not at all** | | | | | □  **a little** | | | | | | | □  **quite well** | | | | | □  **completely** | | | |
| The next questions are about **lost time** because of your headaches. | | | | | | | | | | | | | | | | | | | | | | | | | | | |
| **58** | On how many days **in the last 3 months** could you not go to work or school because of your headaches?  (please enter the number of days missed completely) | | | | | | | | | | | | | | | | | | | | | | | | | _____ | |
| **59** | On how many days **in the last 3 months** could you do **less than half** your usual amount in your job or schoolwork because of your headaches?  (please enter the number of days; do **not** include days you counted in question 58 where you missed work or school) | | | | | | | | | | | | | | | | | | | | | | | | | _____ | |
| **60** | On how many days **in the last 3 months** could you not do any household work because of your headaches?  (please enter the number of days lost completely) | | | | | | | | | | | | | | | | | | | | | | | | | _____ | |
| **61** | On how many days **in the last 3 months** could you do **less than half** your usual amount of household work because of your headaches?  (please enter the number of days; do **not** include days you counted in question 60 where you did not do any household work) | | | | | | | | | | | | | | | | | | | | | | | | | _____ | |
| **62** | On how many days **in the last 3 months** did you miss family, social or leisure activities because of your headaches?  (please enter the number of days) | | | | | | | | | | | | | | | | | | | | | | | | | _____ | |
| The next questions aim to understand how much your headaches affect you **even when you do not actually have an attack**.  Please think carefully about the last day when you did **not** have a headache (not counting today). | | | | | | | | | | | | | | | | | | | | | | | | | | | |
| **63** | When was the last day when you did **not** have a headache?  (please enter the number of days or weeks since your last day **without** headache, or tick the box and go directly to question 67)  (if you had **no headache yesterday**, enter 1 day) | | | | | | | | | | | _____  **days** | | | | | | | _____  **weeks** | | | | | □  **cannot remember** | | | |
| **64** | **On that day**, were you anxious or worried about your next headache episode?  (please tick one box) | | | | | | | | | | | **no** □ **yes** □ | | | | | | | | | | | | | | | |
| **65** | **On that day**, was there anything you could not do or did not do because you wanted to avoid getting a headache?  (please tick one box) | | | | | | | | | | | **no** □ **yes** □ | | | | | | | | | | | | | | | |
| **66** | **On that day**, did you feel **completely free** from all headache-related symptoms?  (please tick one box) | | | | | | | | | | | **no** □ **yes** □ | | | | | | | | | | | | | | | |
| The next questions ask about **willingness to pay for treatment**.  Imagine that there is a treatment you can buy. If you take it, your headaches will no longer bother you. How much would you be willing to pay **every month** for this treatment?  [These questions are not appropriate in all cultures, and may not be appropriate in countries with free or reimbursed health care. If used, they should apply national currency units (NCU). The multiplier X should be such that reasonable expectation of average willingness to pay is matched by NCU 10X.] | | | | | | | | | | | | | | | | | | | | | | | | | | | |
| **67** | Would you pay NCU 5X a month?  (tick one box)  If the answer is no, go to question 68; if the answer is yes, go to question 71. | | | | | | | | | | | **no** □ **yes** □ | | | | | | | | | | | | | | | |
| **68** | Would you pay NCU 2X a month?  (tick one box)  If the answer is no, go to question 69; if the answer is yes, agree an amount between NCU 2X and 5X and go directly to question 75. | | | | | | | | | | | **no** □ **yes** □  **agreed amount: NCU ______** | | | | | | | | | | | | | | | |
| **69** | Would you pay NCU 1X a month?  (tick one box)  If the answer is no, go to question 70; if the answer is yes, agree an amount between NCU 1X and 2X and go directly to question 75. | | | | | | | | | | | **no** □ **yes** □  **agreed amount: NCU ______** | | | | | | | | | | | | | | | |
| **70** | Would you pay anything?  (tick one box)  If the answer is no, go directly to question 75; if the answer is yes, agree an amount between NCU 0 and 1X and go directly to question 75. | | | | | | | | | | | **no** □ **yes** □  **agreed amount: NCU ______** | | | | | | | | | | | | | | | |
| **71** | Would you pay NCU 10X a month?  (tick one box)  If the answer is yes, go to question 72; if the answer is no, agree an amount between NCU 5X and 10X and go directly to question 75. | | | | | | | | | | | **no** □ **yes** □  **agreed amount: NCU ______** | | | | | | | | | | | | | | | |
| **72** | Would you pay NCU 20X a month?  (tick one box)  If the answer is yes, go to question 73; if the answer is no, agree an amount between NCU 10X and 20X and go directly to question 75. | | | | | | | | | | | **no** □ **yes** □  **agreed amount: NCU ______** | | | | | | | | | | | | | | | |
| **73** | Would you pay NCU 50X a month?  (tick one box)  If the answer is yes, go to question 74; if the answer is no, agree an amount between NCU 20X and 50X and go directly to question 74 | | | | | | | | | | | **no** □ **yes** □  **agreed amount: NCU ______** | | | | | | | | | | | | | | | |
| **74** | Would you pay NCU 100X a month?  (tick one box)  If the answer is no, agree an amount between NCU 50X and 100X; if the answer is yes, agree an amount of NCU 100X or more. | | | | | | | | | | | **no** □ **yes** □  **agreed amount: NCU ______** | | | | | | | | | | | | | | | |
| The next three questions are about the effects your headaches have on your relationships, your love life and your choices in family planning.  Please answer no to any that do not apply. | | | | | | | | | | | | | | | | | | | | | | | | | | | |
| **75** | In **the last 3 months**, have your headaches caused difficulties in your love life?  (please tick one box) | | | | | | | | | | | **no** □ **yes** □ | | | | | | | | | | | | | | | |
| **76** | Have your headaches ever caused a long-term relationship or partnership to break down?  (please tick one box) | | | | | | | | | | | □  **no** | | | | | □  **yes, temporarily** | | | | | | | | □  **yes, permanently** | | |
| **77** | Have your headaches affected **your choices** with regard to **family planning**?  (please tick all boxes that apply **because of your headaches**) | | | | | | | | | | | **no**  **yes, I have had fewer children**  **yes, I have avoided having children**  **yes, they have made it harder to conceive**  **yes, I have avoided oral contraception** | | | | | | | | | | | | □  □  □  □  □ | | | |
| The next two questions are for **people with children of school age**.  If they do not apply, please go directly to question 80. | | | | | | | | | | | | | | | | | | | | | | | | | | | |
| **78** | During **the last 3 months**, have **your** headaches caused one or more of your children to miss school?  (please tick one box and, if yes, estimate the total number of missed days) | | | | | | | | | | | **no** □ **yes** □  **total number of days ______** | | | | | | | | | | | | | | | |
| **79** | During **the last 3 months**, have **your** headaches prevented you from taking an interest in your children?  (please tick one box) | | | | | □  **less than once a month** | | | | | | □  **yes, once  or more a month** | | | | | | | □  **yes, once  or more a week** | | | | | □  **yes, every day** | | | |
| The next two sets of questions are for **people with household partners**.  (A household partner may be husband or wife, or an unmarried partner of either gender in a stable relationship.)  If you are not now living with a partner, please go directly to question 87. | | | | | | | | | | | | | | | | | | | | | | | | | | | |
| **80** | During **the last 3 months**, have **your** headaches caused **your partner** to lose time from work?  (please tick one box and, if yes, enter the total number of days lost) | | | | | | | | | | | **no** □ **yes** □  **total number of days ____** | | | | | | | | | | | | | | | |
| **81** | During **the last 3 months**, have **your** headaches caused **your partner** to miss social activities?  (please tick one box and, if yes, enter the total number of occasions missed) | | | | | | | | | | | **no** □ **yes** □  **number of occasions ____** | | | | | | | | | | | | | | | |
| The next five questions are about **your household partner**. We would like to know if your partner has headaches and, if so, how they affect **your** life.  If you are **not** now living with a partner, please go directly to question 87. | | | | | | | | | | | | | | | | | | | | | | | | | | | |
| **82** | Has **your partner** had a headache in the last year?  (please tick one box) | | | | | | | | | | | **no** □ **yes** □  (if no, go directly to question 87) | | | | | | | | | | | | | | | |
| **83** | During **the last 30 days**, on how many days did he/she have a headache?  (enter the number of days between 0 and 30) | | | | | | | | | | | **______ days** | | | | | | | | | | | | | | | |
| **84** | During **the last 3 months**, have **your partner’s** headaches caused **you** to lose time from work?  (please tick one box and, if yes, enter the total number of days lost) | | | | | | | | | | | **no** □ **yes** □  **total number of days ____** | | | | | | | | | | | | | | | |
| **85** | During **the last 3 months**, have **your partner’s** headaches caused **you** to miss social activities?  (please tick one box and, if yes, enter the total number of occasions missed) | | | | | | | | | | | **no** □ **yes** □  **number of occasions ____** | | | | | | | | | | | | | | | |
| **86** | During **the last 3 months**, have **your partner’s** headaches caused difficulties in your love life?  (please tick one box) | | | | | | | | | | | **no** □ **yes** □ | | | | | | | | | | | | | | | |
| The next four series of questions are general, to be **answered by everyone**, with or without headaches. | | | | | | | | | | | | | | | | | | | | | | | | | | | |
| **Body mass index questions**  Your answers to these questions will give an indication of your level of fitness. | | | | | | | | | | | | | | | | | | | | | | | | | | | |
| **87** | What is your weight?  (please enter your weight in kilograms **or** stones and pounds) | | | | | | | | | | | | **______ kg** | | | | | | | | **_____ st ____ lb** | | | | | | |
| **88** | What is your height?  (please enter your height in centimetres **or** feet and inches) | | | | | | | | | | | | **______ cm** | | | | | | | | **_____ ft ____ in** | | | | | | |
| **89** | What is your waist measurement?  (please measure at the level of the umbilicus (navel) and enter the measurement in centimetres **or** inches)  Tick the box if you are pregnant. | | | | | | | | | | | | **______ cm**  **______ in** | | | | | | | | □  **pregnant** | | | | | | |
| **Quality of life questions (WHOQoL-8)**  This set of eight questions, developed by the World Health Organization, are for everybody, whether they have headaches or not. They will help us compare people with headaches and people without.  The questions ask how you feel about your quality of life, health or other areas of your life. Each question has five response options. **Please choose the answer that appears most appropriate by circling the number in the appropriate column.** If you are unsure about which response to give to a question, the first response you think of is often the best one.  Please keep in mind your standards, hopes, pleasures and concerns. We ask that you think about your life **in the last 4 weeks.** | | | | | | | | | | | | | | | | | | | | | | | | | | | |
|  | | | **very poor** | | | | | | **poor** | | | | | **neither poor nor good** | | | | | | **good** | | | **very good** | | | | |
| **90** | How would you rate your quality of life? | | 1 | | | | | | 2 | | | | | 3 | | | | | | 4 | | | 5 | | | | |
|  | | | **very dissatisfied** | | | | | | **dissatisfied** | | | | | **neither satisfied nor dissatisfied** | | | | | | **satisfied** | | | **very satisfied** | | | | |
| **91** | How satisfied are you with your health? | | 1 | | | | | | 2 | | | | | 3 | | | | | | 4 | | | 5 | | | | |
| **92** | How satisfied are you with your ability to perform your daily living activities? | | 1 | | | | | | 2 | | | | | 3 | | | | | | 4 | | | 5 | | | | |
| **93** | How satisfied are you with yourself? | | 1 | | | | | | 2 | | | | | 3 | | | | | | 4 | | | 5 | | | | |
| **94** | How satisfied are you with your personal relationships? | | 1 | | | | | | 2 | | | | | 3 | | | | | | 4 | | | 5 | | | | |
| **95** | How satisfied are you with the conditions of your living place? | | 1 | | | | | | 2 | | | | | 3 | | | | | | 4 | | | 5 | | | | |
|  | | | **not at all** | | | | | | **a little** | | | | | **moderately** | | | | | | **mostly** | | | **completely** | | | | |
| **96** | Do you have enough energy for everyday life? | | 1 | | | | | | 2 | | | | | 3 | | | | | | 4 | | | 5 | | | | |
| **97** | Have you enough money to meet your needs? | | 1 | | | | | | 2 | | | | | 3 | | | | | | 4 | | | 5 | | | | |
| **Subjective wellbeing questions**  These four questions ask how you feel about aspects of your life. Please answer each one on a scale of 0-10, where 0 is “not at all” and 10 is “completely”. | | | | | | | | | | | | | | | | | | | | | | | | | | | |
| **98** | Overall, how satisfied are you with your life nowadays?  (please enter your answer as a number between 0 and 10 where 0 is not at all satisfied and 10 is completely satisfied) | | | | | | | | | | | | | | | | | | | | **(enter 0-10)** | | | | | | |
| **99** | Overall, to what extent do you feel that the things you do in your life are worthwhile?  (please enter your answer as a number between 0 and 10 where 0 is not at all worthwhile and 10 is completely worthwhile) | | | | | | | | | | | | | | | | | | | | **(enter 0-10)** | | | | | | |
| **100** | Overall, how happy did you feel yesterday?  (please enter your answer as a number between 0 and 10 where 0 is not at all happy and 10 is completely happy) | | | | | | | | | | | | | | | | | | | | **(enter 0-10)** | | | | | | |
| **101** | Overall, how anxious did you feel yesterday?  (please enter your answer as a number between 0 and 10 where 0 is not at all anxious and 10 is completely anxious) | | | | | | | | | | | | | | | | | | | | **(enter 0-10)** | | | | | | |
| **The questionnaire is now complete. Thank you very much for your time.** | | | | | | | | | | | | | | | | | | | | | | | | | | | |

| **This section is only for respondents in the validation sub-sample.** | | |
| --- | --- | --- |
| **201** | Physician-diagnosis of most bothersome headache (if made) | **____________________________** |
| **202** | Physician-diagnosis of other headache 1 (if made) | **____________________________** |
| **203** | Physician-diagnosis of other headache 2 (if made) | **____________________________** |
| **204** | Physician-diagnosis of other headache 3 (if made) | **____________________________** |
