# Supplementary material for: Diagnosis, prevalence estimation and burden measurement in population surveys of headache: presenting the HARDSHIP questionnaire
Source: J Headache Pain. 2014 Jan 8;15(1):3. doi: 10.1186/1129-2377-15-3 (PMC3906903; doi:10.1186/1129-2377-15-3)
Supplement: Additional file 2 — Diagnostic algorithm. [file 1129-2377-15-3-S2.pdf]

Lifting The Burden

in official relations with
the World Health Organization

The Global Campaign against Headache

Diagnostic algorithm for headache according to the diagnostic questions in the HARDSHIP questionnaire

Responses to the diagnostic questions of the HARDSHIP questionnaire, for each bothersome headache separately identified by the participant, should be entered into this algorithm.

The algorithm must be applied from the beginning in each case.

Begin here:

Flags for manual review	HARDSHIP question	Response	Value	Action
	14	Headache on ≥15 days/month	0	Continue with migraine algorithm
			1	Continue below
	18	Acute medication on ≥10 days/month	0	Not medication-overuse headache (MOH); manually review for final diagnosis
			1	Possible MOH; manually review for final diagnosis

Migraine algorithm	HARDSHIP question	Response	Value	Calculate 1st intermediate score	Calculate 2nd intermediate score	Final score and diagnosis
B	21/23	< 4 h	0			0
		> 3 d (72 h)	0			0
		≥ 4 h and ≤ 3 d (72 h)	1			1
C	24	Not bad	0	24+25+26+27 ≥ 2<br>24+25+26+27 < 2		1<br>0
		Quite bad	1			
		Very bad	1			
	25	Pulsating/throbbing yes	1			
		Pressing/tightening yes	0			
	26	One side	1			
		Both sides	0			
	27	Worsened by physical activity yes/no	1/0			
D	29	Nausea yes/no	1/0	N+V ≥ 1: 1<br>N+V = 0: 0	n/v+pt/pn ≥ 1<br>n/v+pt/pn = 0	1<br>0
	30	Vomiting yes/no	1/0			
	31	Photophobia (pt) yes/no	1/0	Pt+pn = 2: 1<br>Pt+pn ≤ 1: 0		
	32	Phonophobia (pn) yes/no	1/0			
					B+C+D = 3	**Migraine**
					B+C+D < 3	Not migraine, go to **TTH algorithm**

TTH algorithm	HARDSHIP question	Response	Value	Calculate 1st intermediate score	Calculate 2nd intermediate score	Final score and diagnosis
B	21/23	< ½ h	0			0
		> 7 d	0			0
		≥ ½ h and ≤ 7 d (168 h)	1			1
C	24	Not bad	1	24+25+26+27 ≥ 2<br>24+25+26+27 < 2		1<br>0
		Quite bad	1			
		Very bad	0			
	25	Pulsating/throbbing yes	0			
		Pressing/tightening yes	1			
	26	One side	0			
		Both sides	1			
	27	Worsened by physical activity yes/no	0/1			
D	29	Nausea yes/no	1/0	N+V ≥ 1: 0<br>N+V = 0: 1	n/v+pt/pn = 2<br>n/v+pt/pn < 2	1<br>0
	30	Vomiting yes/no	1/0			
	31	Photophobia (pt) yes/no	1/0	Pt+pn ≤ 1: 1<br>Pt+pn = 2: 0		
	32	Phonophobia (pn) yes/no	1/0			
					B+C+D = 3	**TTH**
					B+C+D < 3	Not TTH, go to **probable migraine algorithm**

Probable migraine algorithm	HARDSHIP question	Response	Value	Calculate 1st intermediate score	Calculate 2nd intermediate score	Final score and diagnosis
B	21/23	< 4 h	0			0
		> 3 d (72 h)	0			0
		≥ 4 h and ≤ 3 d (72 h)	1			1
C	24	Not bad	0	24+25+26+27 ≥ 2<br>24+25+26+27 < 2		1<br>0
		Quite bad	1			
		Very bad	1			
	25	Pulsating/throbbing yes	1			
		Pressing/tightening yes	0			
	26	One side	1			
		Both sides	0			
	27	Worsened by physical activity yes/no	1/0			
D	29	Nausea yes/no	1/0	N+V ≥ 1: 1<br>N+V < 1: 0	n/v+pt/pn ≥ 1<br>n/v+pt/pn < 1	1<br>0
	30	Vomiting yes/no	1/0			
	31	Photophobia (pt) yes/no	1/0	Pt+pn = 2: 1<br>Pt+pn ≤ 1: 0		
	32	Phonophobia (pn) yes/no	1/0			
					B+C+D = 2	**Probable migraine**
					B+C+D < 2	Not pMig, go to **probable TTH algorithm**

**Probable TTH algorithm**	**HARDSHIP question**	**Response**	**Value**	**Calculate 1st intermediate score**	**Calculate 2nd intermediate score**	**Final score and diagnosis**
B	21/23	< ½ h	0			0
		> 7 d	0			0
		≥ ½ h and ≤ 7 d (168 h)	1			1
C	24	Not bad	1	24+25+26+27 ≥ 2<br>24+25+26+27 < 2		1<br>0
		Quite bad	1			
		Very bad	0			
	25	Pulsating/throbbing yes	0			
		Pressing/tightening yes	1			
	26	One side	0			
		Both sides	1			
	27	Worsened by physical activity yes/no	0/1			
D	29	Nausea yes/no	1/0	N+V ≥ 1: 0<br>N+V = 0: 1	n/v+pt/pn = 2<br>n/v+pt/pn < 2	1<br>0
	30	Vomiting yes/no	1/0			
	31	Photophobia (pt) yes/no	1/0	Pt+pn ≤ 1: 1<br>Pt+pn = 2: 0		
	32	Phonophobia (pn) yes/no	1/0			
					B+C+D = 2	**Probable TTH**
					B+C+D < 2	Not pTTH, **undetermined headache**

Repeat the algorithm for the next most bothersome headache when another headache has been identified by the participant.
